# Basement membrane collagens and disease mechanisms

**DOI:** 10.1042/EBC20180071

**Published:** 2019-08-06

**Authors:** Anna Gatseva, Yuan Yan Sin, Gaia Brezzo, Tom Van Agtmael

**Affiliations:** 1 Institute of Cardiovascular and Medical Sciences, University of Glasgow, University Avenue, Glasgow G12 8QQ, United Kingdom; 2 Centre for Discovery Brain Sciences, Medical School, University of Edinburgh, Edinburgh EH16 4SB, United Kingdom

**Keywords:** collagen, extracellular matrix, genetics, model organisms, molecular basis of health and disease, molecular mechanisms

## Abstract

Basement membranes (BMs) are specialised extracellular matrix (ECM) structures and collagens are a key component required for BM function. While collagen IV is the major BM collagen, collagens VI, VII, XV, XVII and XVIII are also present. Mutations in these collagens cause rare multi-systemic diseases but these collagens have also been associated with major common diseases including stroke. Developing treatments for these conditions will require a collective effort to increase our fundamental understanding of the biology of these collagens and the mechanisms by which mutations therein cause disease. Novel insights into pathomolecular disease mechanisms and cellular responses to these mutations has been exploited to develop proof-of-concept treatment strategies in animal models. Combined, these studies have also highlighted the complexity of the disease mechanisms and the need to obtain a more complete understanding of these mechanisms. The identification of pathomolecular mechanisms of collagen mutations shared between different disorders represent an attractive prospect for treatments that may be effective across phenotypically distinct disorders.

## Introduction

Basement membranes (BMs) are specialised extracellular matrix (ECM) structures that compartmentalise tissues, provide structural support and influence cell behaviour and signalling. BMs underlie epithelial and endothelial cells, surround smooth muscle, fat and Schwann cells, and occur in the synaptic cleft. They also link cells with the interstitial matrix which contains fibrillar collagens such as collagen I. BMs contain approximately 60–200 proteins and the composition of individual BMs differs to provide different biomechanical and biochemical properties to support their individual functions [1].

Vertebrates express 28 types of collagen, which are divided into classes based on protein domain structure and supramolecular assembly including fibrillar (e.g. collagen I), network forming (collagen IV), beaded microfibril (collagen VI), multiplexin (e.g. collagen XV and XVIII) and FACIT (fibril-associated collagens with interrupted triple helices, e.g. collagen VII and XVII) collagens. All collagens contain a triple helical collagen domain consisting of a Gly-X-Y repeat in which every third residue is a glycine and X-Y can be any amino acid. Collagens are folded within the endoplasmic reticulum (ER) and their secretion can require enlargement of COPII vesicles through TANGO1 (transport and Golgi organisation 1) and HSP47 (heat shock protein 47) proteins [2].

BM collagens are associated with a wide variety of diseases for which there is a need for treatments. Recent advances in elucidating disease mechanisms and gene/cell therapy-based approaches has identified therapeutic targets and guided proof-of-concept therapies. Here, we will provide a brief overview of recent progress in mechanisms of disease caused by mutations in BM collagens, and development of therapeutic strategies. For more in depth reviews on collagens and their diseases we refer the reader to the following: collagen IV [3–5], collagen VI [6], collagen VII and XVII [7,8], collagen XV and XVIII [9].

## Collagen IV

Collagen IV is the most abundant structural BM component and is essential for BM integrity but not initial BM formation [10]. Vertebrates express six collagen IV α chains (α1(IV)–α6(IV)), encoded by the *COL4A1*–*COL4A6* genes, forming three networks: α1α1α2(IV), α3α4α5(IV), and α5α5α6(IV). While α1α1α2(IV) is expressed in nearly every BM, α3α4α5(IV) and α5α5α6(IV) expression is more restricted [5]. Collagen IV alpha (α) chains contain a N-terminal 7S domain, a central collagen domain with approximately 20 interruptions, and a C-terminal NC1 domain, which initiates collagen folding in the ER. Following secretion, collagen IV molecules form a network in the BM that interacts with integrins, discoidin domain receptors (DDR) and G protein-coupled receptors [3,11].

### Mutations affecting α3α4α5(IV) and α5α5α6(IV)

*COL4A3*–*COL4A5* mutations cause glomerular BM (GBM) defects leading to Alport syndrome (AS) (OMIM **#** 301050, **#** 203780, **#** 104200), which causes renal disease, deafness and eye pathology [3]. AS now also covers thin BM nephropathy and familial benign haematuria phenotypes [12], while deletions spanning *COL4A5* and *COL4A6* cause AS with diffuse leiomyomatosis (OMIM **#** 308940). The production of auto-antibodies against the NC1 domain of α3(IV) underlies the autoimmune disorder Goodpasture syndrome [3]. Besides AS, *COL4A3/COL4A4* mutations are associated with kidney disorders including diabetic kidney disease [13], focal segmental sclerosis [14] and steroid-resistant nephrotic syndrome [15], while *COL4A5/COL4A6* mutations affect axogenesis in zebrafish [16], and *COL4A6* mutations can cause non-syndromic hearing loss [17], indicating a growing role of these mutations in disease.

**Figure 1:**
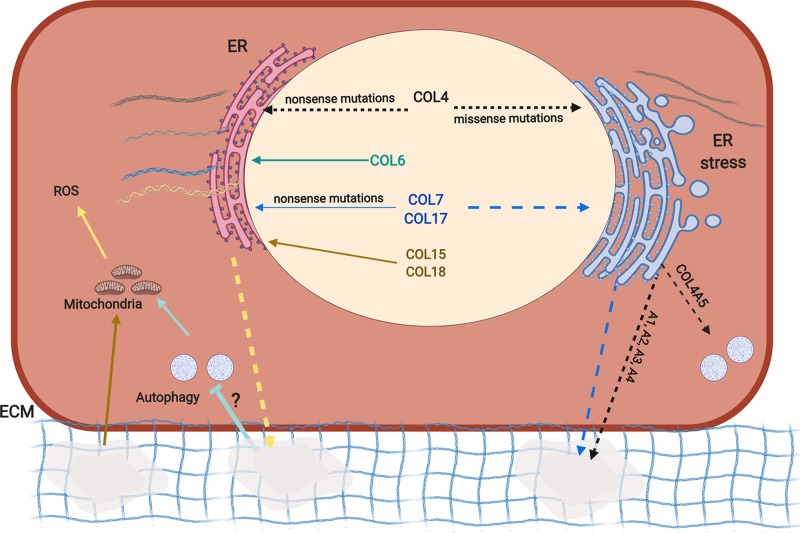
Overview of disease mechanisms caused by mutations in BM collagens Collagen IV (COL4, black arrows) proteins harbouring nonsense mutations are processed in the ER, resulting in reduced secretion of proteins that are incorporated in the ECM, causing matrix defects (indicated by holes). Missense mutations in collagen IV and VII (COL7) can result in their ER retention and ER stress, and subsequent reduction in secretion (dashed arrows). *COL4A5* mutations can also induce autophagy. Mutant collagen IV may also be incorporated in the ECM, resulting in BM defects. Nonsense mutations in collagen VI (COL6), VII (COL7), XVII (COL17), XV (COL15) and XVIII (COL18) (solid arrows) do not result in ER retention but rather in reduced incorporation in the ECM (yellow dashed arrow). Matrix defects resulting from COL6 mutations (light green) lead to failure to induce autophagy (via an as yet unknown mechanism) and result in mitochondrial defects and production of reactive oxygen species (ROS). Matrix defects, resulting from COL15 deficiency (brown arrow) also cause mitochondrial defects and ROS production.

Autosomal dominant AS due to *COL4A3/COL4A4* mutations is milder compared with autosomal recessive or X-linked AS (due to *COL4A5* mutations), and nonsense mutations cause more severe disease compared with missense mutations, the majority of which affect the glycine residue in the Gly-X-Y repeat [3]. Reduced levels or absence of α3α4α5(IV) and associated GBM defects (Figure 1) is a major causative mechanism. Induced α5α5α6(IV) expression in *Col4a3^−/−^*mice (Table 1), a well-established model of AS, reduced disease severity [18] but mosaic α5α5α6(IV) expression in patients was not associated with improved outcome [19]. Similarly, X-linked AS patients exhibited persistent α1α1α2(IV) expression [3]. These data underscore the specific nature of the networks and potential species differences. Interestingly, a laminin β 2 missense variant which was not pathogenic of itself in mice, increased progression to kidney failure in *Col4a3^−/−^* mice and proteinuria in female *Col4a5*^+/−^ mice, supporting the hypothesis that GBM components can act as genetic modifiers [20].

**Table 1:** Frequently used animal models of collagen-related genetic diseases

BM component	Affected gene	Animal model	Disease phenotype (or human equivalent)	References
Collagen IV	*Col4a1*	Mouse missense mutations	Cerebrovascular disease intracerebral haemorrhage Kidney disease Myopathy Eye defects HANAC syndrome	[21–24]
	*Col4a1/ Col4a2 double null*	Mouse	Embryonically lethal, growth retardation,vascular defects	[10]
	*Col4a1 (Cg25c) and Col4a2 (Vkg)*	*Drosophila missense and loss of function mutation*	Intestinal defects, myopathy	[25]
	*emb-9; let-2 (Cola4a1, Col4a2)*	*Caenorhabditis elegans misssense mutations*	Embryonically lethal	[26]
	*Col4a2*	Mouse missense mutations	Cerobrovascular, ocular, renal and muscle defects	[21]
	*Col4a3*	Mouse knockout and missense mutation	Autosomal recessive and dominant AS	[27,28,29]
	*Col4a3 & Col4a4 double null*	Mouse	Juvenile form of AS	[30]
	*Col4a4*	Mouse missense mutation	Autosomal recessive AS	[31]
	*Col4a5*	Mouse knockout and nonsense mutation	X-linked AS	[32,33]
	*Col4a5*	Zebrafish in-frame deletion	Defective retinal axon guiding	[34]
	*Col4a6*	Zebrafish In-frame deletion	Defective axon guiding, cerebellar granule cells defects	[16]
Collagen VI	*Col6a1*	Mouse knockout, heterozygous in frame deletion	Bethlem myopathy. Mitochondrial dysfunction, defective autophagy, fibre necrosis and osteoarthritis, abnormal collagen fibrillogenesis, CNS defect	[35,36]
		Zebrafish morpholino knockdown	Bethlem myopathy, UCMD	[37]
		Zebrafish knockdown	Bethlem myopathy, UCMD, myosclerosis	[38]
	*Col6a3*	Mouse in-frame deletion	Dominant mild myopathy with decreased muscle mass	[39]
		Zebrafish knockdown, in frame deletion	Bethlem myopathy (knockdown), Ullrich syndrome (in frame deletion)	[37,40]
	*Col6a4*	Zebrafish knockdown	Abnormal motoneuron axon growth	[38]
Collagen VII	*Col7a1*	Mouse knockout hypomorph mutation	Recessive dystrophic epidermolysis bullosa	[41,42]
Collagen XV	*Col15a1*	Mouse	Mild skeletal myopathy Cardiomyopathy Vascular dysfunction Defects in nerve development and myelination	[43]
		Drosophila hypomorph mutant: piggybac transposon	Neuronal function defects, cardiomyocyte, skeletal muscle defects	[44,45]
		Zebrafish morpholino knockdown of Col15a1a; Col15a1b knockdown	Defective notochord and muscle development; motor axon guidance defects and muscle atrophy	[46,47],
Collagen XVII	*Col17a1*	Mouse knockout	Non-Herlitz epidermolysis bullosa, growth retardation, enamel hypoplasia	[48]
		Zebrafish col17a1a knockdown; Col17a1b knockdown	Junctional epidermolysis bullosa (Col17a1a); neuronal defect (Col17a1b)	[49]
Collagen XVIII	*Col18a1*	Mouse Col18a1 knockout	Knobloch syndrome; human pigment dispersion syndrome, hydrocephalus, kidney defect, adipocyte differentiation defect-metabolic defect	[50]
		Col15a1 and Col18a1 knockout		[51]
		Col18a1 isoform-specific knockout		[52]
	*cle-1 (Col18)*	*C. elegans*	Defects in cell and axon migration and neuromuscular synapse function	[53]

Due to space limitations, only the original references describing the animal model could be included. HANAC (hereditary angiopathy with nephropathy aneurysm and cramps), CNS (central nervous system) UCMD (Ullrich congenital muscular dystrophy)

Reduced α3α4α5(IV) GBM levels cause matrix defects, remodelling and fibrosis in AS. Recent evidence from *Col4a3*^−/−^ mice supports a central role for mechanical strain [54] on the GBM in this process as it causes endothelin expression in mesangial cells, which results in endothelin A receptor expression in glomeruli. This leads to mesangial cell invasion [55], expression of laminin α 2-chain containing laminins, and focal adhesion kinase activation in podocytes, producing an inflammatory state and matrix metalloproteinase (MMP) activation [56], indicating matrix remodelling. In part, the fibrosis and MMP activation is influenced by LOXL2 (lysyl oxidase-like 2) collagen cross-linking activity [54], matrix-cell signalling via integrins α1β1 and α2β1, as well as DDR receptors [3,57].

The pathomolecular mechanisms of dominant AS can also include primary intracellular responses to expressing dominant mutations as a *Col4a3* glycine mutation in cultured podocytes and *Col4a3* knockin mice caused ER stress, a stress response activated by misfolded protein accumulation (Figure 1) [29]. Similarly, *COL4A5* glycine mutations can induce ER stress and activate autophagy in patient fibroblasts [58] (Figure 1). Intracellular signalling involving STAT3 and TGFβ1 [59,60], which leads to fibrosis, have also been implicated but how they are activated remains less clear. Similarly, the relative contribution of ER stress to dominant AS remains unknown, but it may influence disease expressivity, similar to *COL4A2*-associated stroke [61].

### Mutations affecting α1α1α2(IV)

The role of *COL4A1/COL4A2* in human disease was identified through analysis of mice with *Col4a1/Col4a2* missense mutations (Table 1) [21–23]. These mutations cause a dominant multisystemic disorder including cerebral small vessel disease, intracerebral haemorrhage (ICH), glomerular and tubular kidney phenotypes, eye defects and myopathy [24,62,63] (OMIM 120130, 120090). The location and nature of mutations affects clinical outcome with mutations affecting the CB3 region of α1(IV) causing the clinical subentity HANAC (hereditary angiopathy, nephropathy, aneurysm and cramps) syndrome, [63] and those in the miRNA-29 binding site of the 3′-UTR of *COL4A1* the ischaemic small vessel disease PADMAL (pontine autosomal dominant microangiopathy with leukoencephalopathy) [64]. In mice, *Col4a1* mutations affecting the X or Y residue of the Gly-X-Y repeat are less severe compared with glycine mutations [23], as are *Col4a2* mutations [4]. Importantly, data from patients, mice and *Caenorhabditis elegans* indicate that disease outcome is influenced by genetic and environmental modifiers such as matrix proteins, vaginal birth and exercise [4,65]. The vast majority (approximately 80%) of *COL4A1/4A2* mutations are missense mutations but nonsense or 3′-UTR mutations also occur, supporting pathogenicity of altered levels [4,64]. Interestingly, common variants in *COL4A1/COL4A2* are risk factors for major vascular disease such as ICH [66] and coronary artery disease [67], but the proportion of patients in which these mutations occur remains unclear. Insight is emerging regarding the cellular origin of the phenotypes whereby both endothelial cell and smooth muscle cells contribute to ICH [68], vascular defects to the myopathy [69] and the lens to the eye disease [70].

Several non-mutually exclusive pathomolecular disease mechanisms have been proposed: intracellular retention of misfolded protein causing ER stress; reduced collagen IV incorporation in the BM and/or incorporation of mutant protein (Figure 1)**.** Structural matrix defects alongside reduced α1α1α2(IV) levels in the BM, which can be associated with MMP activity and fibrosis [24,68], are almost universally observed [4,61,22,23,62], and data from patients with nonsense mutations [4] indicate matrix defects can be sufficient to cause cerebrovascular disease. However, the mechanism for dominant missense mutations is potentially more complicated given the possible contribution of ER stress due to collagen retention. In a family with a *COL4A2* mutation, we uncovered that ER stress and not matrix defects was associated with disease [61], while in mice ICH severity correlated with levels of ER retention [68]. However, not every glycine mutation induces ER retention and stress [4]. These data suggest that ER stress can have a modifier effect for ICH and that cellular consequences and, potentially, mechanisms are mutation dependent. Moreover, our analysis of renal disease in mice revealed that the glomerulopathy was associated with matrix defects but tubular disease with ER stress [62,71], indicating cell/tissue-specific disease mechanisms [62,71]. This was subsequently confirmed for myopathy and ICH [72]. Given this mechanistic interplay of mutation-, cell- and tissue-dependent mechanisms, delineating the relative contribution of cellular consequences and matrix defects to the different phenotypes for different mutations will be informative and important.

## Collagen VI

Vertebrates express six collagen VI α chains, encoded by genes *COL6A1*–*COL6A6*, which form beaded microfibrils that anchor BMs to the interstitial matrix. The major collagen VI monomer is α1α2α3(VI) but the roles of α4(VI), α5(VI) or α6(VI) remain less clear [73]. The central collagen domain of collagen VI is flanked by a globular N- and C-terminal domains, containing motifs with homology to von Willebrand factor type A domains. Within the ER, α chains form antiparallel dimers and then tetramers, which after secretion generate beaded microfibrils [6].

*COL6A1/COL6A2/COL6A3* mutations cause severe Ullrich congenital muscular dystrophy (UCMD) and milder Bethlem myopathy, which can be inherited as autosomal dominant or recessive disorders, but collagen VI mutations can also affect the skin and tendon [6]. The role of *COL6A4–COL6A6* in disease remains unclear [6] but in mice increased *Col6a4* levels causes Hirschsprung’s disease type defects [74]. Similar to collagen IV diseases, genetic and environmental modifiers contribute to the large variability in clinical presentation.

Collagen VI diseases are associated with reduced levels or aberrant incorporation into the matrix of α1α2α3(VI) fibrils with a complete absence causing severe forms of disease [75] (Figure 1). Some heterozygous premature termination codon (PTC) mutations in *COL6A1* cause Bethlem myopathy [76] but they are non-pathogenic when they occur in *COL6A2/COL6A3* [6]. There is also clustering of dominant glycine mutations within the N-terminal end and recessive glycine mutations in more C-terminal regions, suggesting functional domains in these regions [6,77]. The N-terminal dominant glycine mutations and in-frame deletions often affect tetramer and microfibril assembly [78], while more C-terminal in-frame deletions and recessive glycine mutations can affect trimer formation [79]. ER stress has not been reported, and these data strongly support absence of collagen VI or presence of mutant collagen VI in the ECM as being causal.

Analysis of *Col6a1^−/−^*mice (Table 1) that develop Bethlem myopathy provided key insights into the disease mechanisms, including a role for neuromuscular junction and muscle stem cell defects [80,81]. Key steps in the pathomolecular mechanism includes the failure to induce autophagy, associated with reduced Bnip3 (Bcl2 Interacting Protein 3) and Beclin levels, to remove defective mitochondria [82]. This affects the permeability transition pore, reducing ATP synthesis [83], and causing mitochondrial dysfunction and reactive oxygen species (ROS) generation. Further insight is needed into how matrix defects cause these cellular defects and the interplay between them (Figure 1).

## Collagen VII

Collagen VII is encoded by the *COL7A1* gene, and three pro-α1(VII) α chains interact to form α1α1α1(VII) monomers which contain a central collagen domain, with 19 interruptions in the Gly-X-Y sequence, flanked by N-terminal (NC-1) and C-terminal (NC-2) domains [8]. Secreted pro-collagen VII molecules form antiparallel dimers, which are cleaved at the NC-2 domain by bone morphogenic protein 1 during their aggregation to form anchoring fibrils [84]. These anchoring fibrils mediate dermal–epidermal adhesion via binding of collagen VII to laminin-332 and collagen IV in the BM, and collagen I in the interstitial matrix [8]. Auto-antibodies against epitopes in the NC-1 domain cause the autoimmune skin blistering disorders epidermolysis bullosa acquisita (EBA) [85] and bullous systemic lupus erythematosus [86].

Mutations in *COL7A1* cause dystrophic epidermolysis bullosa (DEB) (OMIM # 131750 # 226600) whereby the generally milder dominant DEB (DDEB) and non-syndromic congenital nail disorder-8 (OMIM # 607523) are due to missense mutations. The generally more severe recessive form of DEB (RDEB) is due to homozygous or compound heterozygous *COL7A1* mutations, most frequently leading to nonsense mediated decay (NMD) and absence of collagen VII [8]. This causes blisters, wounding, inflammation, reduced myofibroblast removal and fibrosis. Collagen VII also plays a direct role in would healing by affecting keratinocyte migration via organising laminin-332 and impacting integrin α6β4 signalling, and supporting fibroblast migration and controlling their cytokine production [87]. Disease severity may also be subject to genetic modifiers as increased severity has been associated with a functional variant in *MMP1* [88], which degrades collagen VII, causing increased degradation and increased RDEB severity. However, this association has been questioned [89].

The pathomolecular mechanism of RDEB is the absence of α1α1α1(VII) in the matrix affecting anchoring fibrils, weakening the BM and causing blistering (Figure 1). However, dominant missense mutations can cause ER retention, suggesting that α1α1α1(VII) production, secretion and storage are altered in patients [90]. However, its effect on ER stress and downstream effects has not been reported.

## Collagen XVII

Collagen XVII (aka BPAG2, BP180) is a transmembrane homotrimer α1α1α1(XVII), consisting of three α1(XVII) chains that is important for cell–matrix interactions as a component of hemidesmosomes, securing the attachment of epithelial cells to BMs through binding laminin-332 and potentially collagen IV [91]. Collagen XVII plays a role in autoimmune blistering disease as autoantibodies to collagen XVII cause Bullous Pemphigoid (BP), the most common autoimmune blistering skin disease [48] and linear IgA dermatosis [91]. The latter is caused by auto-antigens within the ectodomain of soluble collagen XVII, which can be generated by ADAM (A Disintegrin And Metalloproteinase) -mediated protease cleavage of collagen XVII [92].

A series of elegant papers has recently identified non-structural functions of collagen XVII. This includes a key role in stem cell maintenance/homeostasis as depletion of collagen XVII in skin, which occurs with normal ageing [93], causes stem cells in the hair follicle to terminally differentiate, causing hair loss [94]. An important role in ageing was also uncovered as replenishment of collagen XVII reverses the hyperproliferation of interfollicular epidermis induced with ageing skin [93], and promotes symmetrical division of epidermal stem cells, which outcompete asymmetrically dividing stem cells. This reversed skin ageing and improved wound healing [95].

*COL17A1* mutations cause junctional epidermolysis bullosa (OMIM # 226650), which can also be caused by mutations in the laminin genes *LAMA3, LAMB3, LAMC2* and integrin genes *ITGA3, ITGA6* and *ITGB4* [7,96,97], and is characterised by dermal–epidermal separation leading to skin blistering, and epithelial recurrent erosion dystrophy, alopecia and nail dystrophy [96]. The majority of mutations are nonsense mutations but some glycine missense mutations occur, which cause intracellular retention and thermal instability of the protein [98], indicating protein misfolding. However, it is unclear if this results in ER stress (Figure 1). The reduction or complete absence of collagen XVII in the matrix affects the hemidesmosomes and stability of the dermal BM zone leading to EB (Figure 1), and genotype–phenotype analysis revealed approximately 12–25% of normal protein is required for skin stability [96]. Dominant *COL17A1* missense mutations have recently been identified as a cause of epithelial recurrent corneal erosion dystrophy (OMIM# 122400) [99] but no functional analysis was performed.

## Collagen XV and XVIII

Collagen XV and XVIII are multiplexin (multiple triple-helix domains with interruptions) collagens that are structurally closely related heparan sulphate proteoglycans. The collagen domain of collagen XVIII is flanked by a N-terminal non-collagenous domain containing thrombospondin-1-like and frizzled motifs [100], and a C-terminal trimerisation domain, which harbours the fragment endostatin that shares homology with restin in collagen XV [9]. The variable length of the N-terminal fragment of α1(XVIII) generates three isoforms with different expression patterns and functions [9]. For example, the short isoform is essential for retinal development while the medium and long isoforms are required for hepatocyte survival following injury [101] and adipose tissue formation [102]. Collagen XVIII is embedded into the BM by its C-terminal domain that binds perlecan and laminin, while its N-terminal domain extends into the ECM. Protease cleavage releases the anti-angiogenic fragment endostatin which inhibits endothelial cell migration and tumour growth. However, its efficacy for human cancer remains debated [9]. In addition, endostatin affects processes such as autophagy [103], its absence causes glomerular and tubular kidney defects, and exacerbates nephritis [104], and an endostatin-derived peptide can reduce fibrosis [105]. Elegant recent work using mouse models established that the frizzled motif in the N-terminal domain is required for preadipocyte differentiation and the ability of white adipose tissue to store fat by inhibiting Wnt signalling [102] (Table 1).

*COL18A1* mutations, the vast majority of which are nonsense mutations, cause recessive Knobloch syndrome (OMIM # 267750) that is characterised by eye defects (e.g. myopia, vitreoretinal degeneration, macular abnormalities) and occipital encephalocele. Deficiency of *Col18a1* in mice mimics Knobloch syndrome (Table 1 and Figure 1) but also affects the kidney and fat metabolism [9]. Many vascular defects of Knobloch syndrome, including persistence of the hyaloid vasculature, have been attributed to the absence of the short isoform and endostatin which generates a pro-angiogenic environment, although absence of the thrombospondin domain may also contribute [9,52]. This pro angiogenic environment due to the absence of endostatin, potentially combined with its effects on proteostasis and autophagy [106], are also associated with age-related retinal pigment epithelium degeneration in *Col18a1^−/−^* mice, a hallmark of age-related macular degeneration.

Collagen XV and XVII are structurally similar molecules with anti-angiogenic fragments, although data have challenged that for collagen XV this is mediated via its restin fragment [9,107]. Despite this structural similarity, these collagens have different functions. Collagen XV is highly expressed in tissues such as heart and skeletal smooth muscle, and structural analysis combined with muscle and vascular defects in *Col15a1*^−/−^ deficient mice supports it acts as a spring between the BM and interstitial matrix to protect against contractile forces [108,43]. Investigation of Col15/18-deficient muscle defects in *Drosophila* uncovered they are due to altered integrin activity causing mitochondrial defects, reduced ATP generation and ROS production [44] (Figure 1). Treatment with cyclosporin A or losartan reduced these phenotypes [44], indicating a pathomolecular mechanism shared with *COL6A1* mutations (see above) (Figure 1).

Analysis of zebrafish, which has two *Col15a1* isoforms, *Col15a1a* and *Col15a1b*, suggests a potential developmental origin to this muscle phenotype as *Col15a1a* deficiency causes defective notochord and muscle development [46]. Deficiency of *Col15a1b* leads to motor axon guidance defects and muscle atrophy [47], establishing a role in neuromuscular development, while in mice α1α1α1(XV) contributes to peripheral nerve development and myelination [109]. In line with its high levels of expression in the heart, deficiency of collagen XV causes cardiomyopathy associated with matrix remodelling, defects in vascular permeability and haemodynamics, and increased stiffness [110]. Recent genetic analysis suggested *COL15A1* can act as a modifier of thoracic aortic aneurysm severity [111] and has indicated a potential role in primary open angle glaucoma [112] and patients with Cuticular drusen (CD), a subtype of age-related macular degeneration [113]. These data indicate a growing role for collagen XV in human biology and disease.

## Treatment intervention

**Figure 2:**
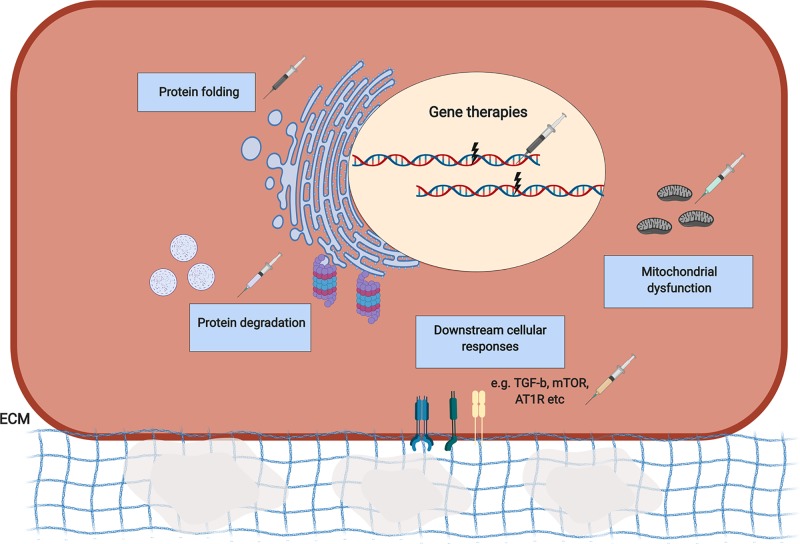
Overview of therapeutic strategies for BM collagen disorders Gene therapy approaches have been implemented to silence disease alleles of *COL6A1* and *COL7A1* mutations using AONs and siRNAs. RNA trans-splicing strategies have also been implemented. Targeted pathomolecular effects of mutations include intracellular retained misfolded proteins and ER stress, which has been used for collagen IV mutations via chemical chaperones to increase protein folding and increase secretion of proteins. Additionally, promoting autophagy and the proteasome may promote degradation of misfolded proteins. Autophagy and mitochondrial defects due to *COL6A1/COL15A1* mutations have also been targeted pharmacologically and through diet. As an example of targeting downstream cellular responses or signalling to modulate disease, TGF-β signalling has been targeted in epidermolysis bullosa due to *COL7A1* mutations. For a more detailed overview of therapeutic strategies, we refer the reader to Table 2. Abbreviation: AON, antisense oligonucleotide.

The identification of underlying disease mechanisms has instigated a plethora of different approaches to modulate disease outcome. Broadly, these strategies can be divided into those that attempt to cure or overcome the genetic defect (gene therapy-based approaches), those that aim to modulate the pathomolecular disease mechanisms or cellular responses of mutations, and those that target further downstream targets (Figure 2 and Table 2). While an in-depth overview is beyond the scope of this review, a few examples of each class are discussed below.

**Table 2:** Mechanism-based therapeutic strategies for collagen-related disease

Gene	Disease	Mechanism-target	Treatment	References
*COL4A1, COL4A2, COL4A5*	Col4a1 disease and AS	ER retained protein, ER stress pathway	Chemical chaperones, e.g. 4-phenylbutyrate (4PBA) to reduce ER stress and increase secretion of correctly folded protein	[58,61,68,71]
*COL4A5*	AS	Blood pressure by targeting renin–angiotensin system	Angiotensin-converting enzyme inhibitors, e.g. ramipril	* [114]
			Angiotensin II type 1 receptor blockers, e.g. losartan	*[115]
		Fibrosis-Transforming growth factor-β 1 (TGF-β), Connective tissue growth factor, miR-21	HMG-CoA-reductase inhibitor (cerivastatin)	[116]
			Vasopeptidase inhibitor AVE7688	[117]
			Anti-miR-21 oligonucleotides	[118]
		Oxidative stress, inflammation and fibrosis: Nrf2	Nrf2 activator, e.g. bardoxolone methyl (BARD)	*[119]
		STAT3 signalling	STAT3 inhibitor, e.g. stattic	[59]
		Functional correction	Gene therapy: restoration of network proof of concept	[120]
			Cell therapy Bone marrow-derived stem cells	[121]
			Amniotic fluid stem cells	[122]
*COL6A1*	Bethlem myopathy, Ullrich congenital muscular dystrophy	Reactivation of autophagy	mTOR inhibitor, e.g. Rapamycin	[82]
			Low protein diet	*[123]
			Spermidine	[124]
		Mitochondrial defect: opening of Mitochondrial permeability transition pore (mPTP)	Cyclosporin A Cyclophilin inhibitor, e.g. NIM811, Debio25 (alisporivir)	*[125,83,37,126,127]
		Metabolic defects	Adiponectin	[128]
		Functional correction Collagen VI-producing cells	Cell therapy: fibroblast grafting	[81]
			Adipose-derived stem cell transplant	[129]
		Dominant negative mutation	Gene silencing with AONs or siRNAs	[130,131,132,133]
		Splice mutations	AON-mediated exon skipping	[134]
*COL7A1*	DEB	Wound healing	Injecting fibroblast cells	*[135]
			Grafting revertant mosaicism skin-keratinocytes	*[136]
			Genome editing patient-derived IPSC cells and transplant	[137]
			Mesenchymal stromal cell therapy transplant	*[138]
			Human placental‐derived stem cell transplant	[139]
		Functional correction	Exon skipping	[140]
	RDEB	Functional correction	*Ex vivo* TALEN gene editing	[141]
			*Ex vivo* CRISPR Genome editing keratinocytes	*[142]
			RNA trans-splicing	[143]
			Polymer-mediated cDNA delivery	[144]
			*Ex vivo* retroviral transduction	[145]
			AON-mediated exon skipping	[146]
			Read through of Premature termination codons (PTCs)	See review [147]
		Fibrosis: TGF-β	Angiotensin II type 1 receptor antagonist: losartan	[148]
	DDEB	Functional correction	Allele-specific silencing via siRNA	[149]
			Gene editing using NHEJ to knockout mutant allele	[146,150]
		Deficient collagen VII levels in ECM	Protein replacement therapy	[151,152]
*COL15A1/COL18A1*	Muscular defect	Mitochondrial defect (opening permeability transition pore) and ROS production	Cyclosporine A Angiotensin II type 1 receptor antagonist, e.g. losartan	[44]

Clinical trials are indicated by *. Abbreviation: AON, antisense oligonucleotide.

### Gene therapy approaches

Gene therapy approaches are attractive as they are independent of disease mechanism and provide an actual cure. For missense mutations, allelic silencing using siRNA or gapmer antisense oligonucleotides (AONs) has been successfully applied to *COL6A1* and *COL7A1* mutations, with AONs affecting wild-type allele expression to a lesser extent [132,133], although this requires mutation specific compounds. Splice mutations in *COL6A1/COL7A1* have also been countered using AONs that mediate exon skipping [134], but trans-splicing to obtain wild-type protein expression can be applied to splicing and PTC-generating mutations [143].

Skin disorders are particularly amenable to *ex vivo* genome editing using CRISPR-Cas9 from patient-derived induced pluripotent stem cells combined with skin grafting. This has been successful for junctional EB due to laminin mutations [153,145]. While, stem cell treatment employing iPSCs for some tissues/diseases may have its challenges, enormous progress has been made, making this an exciting approach [154]. For RDEB clinical trials employing *ex vivo* retroviral transduction of patient keratinocytes or fibroblasts to induce collagen VII expression followed by grafting or fibroblast injection, respectively, has shown promise [145,155]. These represent mutation-independent approaches for BM collagens.

### ER stress and other disease mechanisms

Dominant missense mutations occur across collagen types and resultant protein misfolding, ER retention and ER stress represent potential convergent disease mechanisms. Promoting protein degradation, as performed for collagen X mutations [156] or protein folding through chaperones are potential strategies to address this. We and others established that the chemical chaperone 4-PBA reduces ER stress due to *COL4A2* or *COL4A5* mutation in patient cells [58,61]. Importantly, 4-PBA reduced cerebrovascular disease severity in mice carrying *Col4a1* missense mutations at the C-terminal end of the collagen domain that cause intracellular retention [68,71], as a preventative approach and as a treatment for established disease [71]. However, we also established that 4-PBA was not effective for eye and kidney disease due to the same glycine mutation and that PBA increased secretion of collagen IV and weakened the BM [71]. Therefore, 4-PBA treatment may be contra-indicative for matrix-related phenotypes or missense mutations that do not cause ER stress [71]. This was subsequently confirmed for Col4a1 myopathy in mice carrying a more N-terminal mutation that does not induce ER stress [72]. Therefore, treatments will need stratification according to the mechanisms of mutations.

Mitochondrial dysfunction and dysregulation of autophagy resulting from collagen VI or XV/XVIII deficiency was ameliorated by dietary and pharmacological treatments when tested in mice and patient-derived cells [82,37,123,126], indicating a convergent mechanism and treatment. Re-instating collagen expression via cell graft or cell transplant represents a different approach and has shown promise for collagen VI and VII [81,145,155,129]. Re-instating *Col6a1* expression also rescued the defective muscle stem cell renewal in *Col6a1^−/−^* mice, which was recalcitrant to autophagy treatment [81], suggesting a combinatorial therapy may be required.

While the above approaches target the intracellular consequences, due to our relatively poor understanding of the matrix defects and the subsequent aberrant matrix–cell signalling, limited progress has been made. An emerging example is the pharmacological inhibition of DDR1 to preserve renal function and reduce renal fibrosis in *Col4a3*^−/−^ mice [157], illustrating the potential power of this approach.

### Targeting downstream effect and treatment of symptoms

Alternatively, interventions have focused on particular disease symptoms or further downstream consequences. For example, inhibitors of the renin–angiotensin system (e.g. ramipril) are used to reduce blood pressure in AS to delay kidney failure [3].

Fibrosis is a common feature of matrix disorders and targeting TGF-β-induced matrix deposition has been undertaken via anti-miR-21 or pharmacological STAT3 inhibition in Alport mice [118,59]. Interestingly, inhibition of TGF-β signalling using losartan was also effective in RDEB, indicating that convergent downstream pathways represent a potential effective target for different phenotypes. Finally, the compound bardoxolone, which reduces inflammation and fibrosis in chronic kidney disease, is currently in a clinical trial for AS [119]. However as bardoxolone increases glomerular filtration rate, which may elevate the stress on the damaged GBM of Alport patients, this approach remains debated [158].

## Outlook

BM collagen disorders are archetypical rare disorders for which treatment development can represent an economic challenge. Therefore, identifying convergent mechanisms and targets represent a very attractive proposition for both industry and patients, as it stands to accelerate treatment development. However, the multi-systemic nature, variable expressivity and disease severity pose a huge challenge. Therefore, there is an urgent need to increase our fundamental understanding of BM biology and acquire in-depth knowledge of disease mechanisms from gene to patient level. This includes, but is not limited to, the identification of genetic modifiers, a complete description of phenotypes in which BM collagens play a role, and establishing the relative contribution of intra/extracellular mechanisms including matrix–cell signalling to disease. In addition, the multi-systemic nature of these diseases with mutation-, cell- and tissue-specific mechanisms needs to be considered. However, while a ‘one-size-fits-all’ therapy is not possible, the identification of convergent aspects does raise the possibility that several diseases or mutation types may be amenable to manipulation of a shared mechanism. The stratified treatments would therefore focus on pathophysiological mechanisms and unite clinically disparate phenotypes.

## Summary

- Basement membrane collagens play an important role in a growing number of Mendelian disorders and common traits, and increasing our knowledge of basement membrane biology and disease mechanisms will help address the need for treatments.
- Mechanistic studies have provided insight into the pathomolecular mechanisms of BM diseases, revealing that intra- and extracellular mechanisms are associated with disease.
- The identification of disease mechanisms shared between distinct diseases raises the potential that several diseases or mutation types may be treated by manipulation of a shared mechanism.
- Disease mechanism-based therapies are being explored using preclinical animal models and several have been taken forward to clinical trials.

## Abbreviations

AON: antisense oligonucleotide
AS: Alport syndrome
BM: basement membrane
DDR: discoidin domain receptor
DEB: dystrophic epidermolysis bullosa
ECM: extracellular matrix
ER: endoplasmic reticulum
GBM: glomerular BM
ICH: intracerebral haemorrhage
iPSC: induced Pluripotent Stem Cell
NC: non-collagenous
MMP: matrix metalloproteinase
OMIM: Online Mendelian Inheritance in Man
PTC: premature termination codon
RDEB: recessive form of DEB
ROS: reactive oxygen species
